# Patient's Perception of Digital Symptom Assessment Technologies in Rheumatology: Results From a Multicentre Study

**DOI:** 10.3389/fpubh.2022.844669

**Published:** 2022-02-22

**Authors:** Johannes Knitza, Felix Muehlensiepen, Yuriy Ignatyev, Franziska Fuchs, Jacob Mohn, David Simon, Arnd Kleyer, Filippo Fagni, Sebastian Boeltz, Harriet Morf, Christina Bergmann, Hannah Labinsky, Wolfgang Vorbrüggen, Andreas Ramming, Jörg H. W. Distler, Peter Bartz-Bazzanella, Nicolas Vuillerme, Georg Schett, Martin Welcker, Axel J. Hueber

**Affiliations:** ^1^Department of Internal Medicine 3, Friedrich-Alexander-University Erlangen-Nürnberg and Universitätsklinikum Erlangen, Erlangen, Germany; ^2^Deutsches Zentrum für Immuntherapie (DZI), Friedrich-Alexander-University Erlangen-Nürnberg and Universitätsklinikum Erlangen, Erlangen, Germany; ^3^Université Grenoble Alpes, AGEIS, Grenoble, France; ^4^Center for Health Services Research, Faculty of Health Sciences, Brandenburg Medical School Theodor Fontane, Rüdersdorf, Germany; ^5^Verein zur Förderung der Rheumatologie e.V., Würselen, Germany; ^6^RheumaDatenRhePort (RHADAR), Planegg, Germany; ^7^Klinik für Internistische Rheumatologie, Rhein-Maas Klinikum, Würselen, Germany; ^8^Institut Universitaire de France, Paris, France; ^9^LabCom Telecom4Health, Orange Labs & Univ. Grenoble Alpes, CNRS, Inria, Grenoble INP-UGA, Grenoble, France; ^10^MVZ für Rheumatologie Dr. Martin Welcker GmbH, Planegg, Germany; ^11^Section Rheumatology, Sozialstiftung Bamberg, Bamberg, Germany; ^12^Division of Rheumatology, Klinikum Nürnberg, Paracelsus Medical University, Nürnberg, Germany

**Keywords:** telemedicine, symptom assessment [MeSH], artificial intelligence, eHealth, diagnostic decision support system (DDSS), rheumatology, mobile app

## Abstract

**Introduction:**

An increasing number of digital tools, including dedicated diagnostic decision support systems (DDSS) exist to better assess new symptoms and understand when and where to seek medical care. The aim of this study was to evaluate patient's previous online assessment experiences and to compare the acceptability, usability, usefulness and potential impact of artificial intelligence (AI)-based symptom checker (Ada) and an online questionnaire-based self-referral tool (Rheport).

**Materials and Methods:**

Patients newly presenting to three German secondary rheumatology outpatient clinics were randomly assigned in a 1:1 ratio to complete consecutively Ada or Rheport in a prospective non-blinded multicentre controlled crossover randomized trial. DDSS completion time was recorded by local study personnel and perceptions on DDSS and previous online assessment were collected through a self-completed study questionnaire, including usability measured with the validated System Usability Scale (SUS).

**Results:**

600 patients (median age 52 years, 418 women) were included. 277/600 (46.2%) of patients used an online search engine prior to the appointment. The median time patients spent assessing symptoms was 180, 7, and 8 min, respectively using online using search engines, Ada and Rheport. 111/275 (40.4%), 266/600 (44.3%) and 395/600 (65.8%) of patients rated the respective symptom assessment as very helpful or helpful, using online search engines, Ada and Rheport, respectively. Usability of both diagnostic decision support systems (DDSS) was “good” with a significantly higher mean SUS score (SD) of Rheport 77.1/100 (16.0) compared to Ada 74.4/100 (16.8), (*p* < 0.0001). In male patients, usability of Rheport was rated higher than Ada (*p* = 0.02) and the usability rating of older (52 years ≥) patients of both DDSS was lower than in younger participants (*p* = 0.005). Both effects were independent of each other. 440/600 (73.3%) and 475/600 (79.2%) of the patients would recommend Ada and Rheport to friends and other patients, respectively.

**Conclusion:**

In summary, patients increasingly assess their symptoms independently online, however only a minority used dedicated symptom assessment websites or DDSS. DDSS, such as Ada an Rheport are easy to use, well accepted among patients with musculoskeletal complaints and could replace online search engines for patient symptom assessment, potentially saving time and increasing helpfulness.

## Introduction

Being confronted with new symptoms, we increasingly turn to the internet first to seek further information (1–3). Besides traditional search engines, an increasing number of dedicated symptom assessment websites and apps exist, that point out diagnostic suggestions and or action advice (4). These patient-facing diagnostic decision support systems (DDSS), often also being referred to as “symptom checkers” (5) are increasingly being used by the general population (1) and rheumatic patients in particular (2, 3, 6, 7). The instant availability of help offered by these digital tools are very appealing to the general public and the low costs make these tools very appealing to politicians and health care systems (1).

DDSS are currently based on very different approaches covering a varying number of disciplines and diagnoses (4, 8). Rheport for example is an online self-referral tool, designed to optimize rheumatology referrals, being based on a fixed questionnaire (9, 10), whereas Ada is an artificial intelligence (AI) and chatbot-based symptom checker covering multiple diagnoses (8, 10). Furthermore, Ada's questions are dynamically chosen and the total number of questions varies depending on the previous answers given.

Due to these different approaches, acceptability and usability might significanty differ between DDSS approaches and not everyone might be able to efficiently use these new tools or appreciate them.

Currently, DDSS evaluation studies largely focus on the evaluation of the diagnostic accuracy (4, 10, 11) and only few studies analyzed the usability and acceptability by their end-users, i.e., actual patients. A recent study conducted by the symptom checker company Ada Health suggests that the majority of patients (511/522, 97.8%) rated the symptom checker Ada as very easy or quite easy to use and would recommend it to a friend or relative (444/520, 85.3%) (12). Importantly, the authors discovered a trend for younger respondents to rate Ada as more helpful. Meyer et al. (13) recently showed that compared with patients who had not previously experienced diagnostic errors (missed or delayed diagnoses: 123/304, 40.5%), patients who had previously experienced diagnostic errors (181/304, 59.5%) were more likely to use a symptom checker (Isabel) to determine where they should seek care (15/123, 12.2 vs. 48/181, 26.5%; *P* = 0.002).

The current literature seems limited to isolated assessments and to our knowledge no study has been carried out yet, directly comparing digital symptom assessment systems to each other and the current “gold standard”, conventional online search engines. To generate real-world-based evidence and allow direct comparison, we initiated a crossover randomized controlled multicentre trial, conducted at three rheumatology centers in Germany, where patients completed two DDSS, with different questioning approaches (Ada and Rheport), consecutively before their regular appointment (10). Furthermore, patients completed a questionnaire to assess previous online symptom assessment and DDSS perception. The results of a first interim analysis (10), focusing on diagnostic accuracy and including 164 patients from the first recruiting center suggested that the majority of patients would recommend both tools to other patients and friends, with a slight preference for Rheport (67.1 vs. 64.0%).

The aim of this analysis was to evaluate patient's previous online assessment experiences, to compare the acceptability, usability, usefulness and potential impact of artificial intelligence (AI)-based symptom checker (Ada) and an online questionnaire-based self-referral tool (Rheport) using the final dataset from all three rheumatology centers.

## Materials and Methods

### Study Design and Participants

A crossover randomized controlled multicentre trial, conducted at three rheumatology centers in Germany (University hospital Erlangen, general hospital Bamberg, private practice Planegg), where adult patients newly presenting to the respective outpatient clinic with musculoskeletal symptoms and unknown diagnosis were included. All patients provided written informed consent and completed both DDSS consecutively at the respective rheumatology center on tablets (Erlangen: Apple iPads, others: Samsung Galaxy tablets) before their regular appointment, with assistance provided if necessary. The DDSS order of completion was randomized by using a computer-generated block randomization whereas each block contains *n* = 100 patients, to exclude a bias caused by previous completion of Ada/Rheport. DDSS completion time was recorded by local study personnel and perceptions on the DDSS and previous online assessment were collected through a self-completed study questionnaire following DDSS completion. Usability as a main outcome was measured using the ten-item System Usability Scale questionnaire (SUS) (14). SUS has been translated and validated in multiple languages and is one of the most established usability questionnaires (15). The SUS score ranges between 0 (worst) and 100 (best), where a score >68 should be considered above average and a score >80 as high (14). Furthermore, SUS values were translated to categories such as “excellent” using the adjective SUS rating scale as previously described by Bangor et al. (16). The questionnaire additionally captured: Time spent using online search engines (minutes), perceived helpfulness of online search and DDSS usage, using a 5-Point Likert-scale (1 = not helpful at all, 5 = very helpful); if patients would recommend the DDSS to friends and other patients (yes/no); and what potential impact DDSS would have made on their decision to see a physician and to worry. The study was approved by the ethics committee of the medical faculty of the university of Erlangen-Nürnberg, Germany (106_19 Bc) and was conducted in compliance with the Declaration of Helsinki. This trial was propspectively registered in the German Clinical Trials Register (DRKS00017642). The primary outcome of the trial, DDSS diagnostic accuracy, will be investigated separately and this study reports all secondary outcome findings, namely patient-perceived usability, acceptance and usefulness. A sample size calculation has been carried out only regarding the primary outcome. An interim analysis, including the first 164 patients from the first recruiting center (University hospital Erlangen) has been previously published (10).

### Description of Diagnostic Decision Support Systems (Ada and Rheport)

Ada (www.ada.com) is an artificial intelligence and app based chatbot. The app covers a broad range of different symptoms and diseases, not being limited to rheumatology (8). More than 15 million symptom assessments have been completed with Ada in 130 countries (17) and its diagnostic accuracy is allegedly superior to other DDSS, nearly equal to general physicians (8). Users are asked for basic health information (sex, age etc.) and then for current symptoms. Depending on the answers given, further questions are asked, so that each symptom assessment is individual. The app suggestions are based on a Bayesian network, which is constantly updated (12). More detailed method descriptions involving authors from Ada can be found in previous publications (8, 12). Once symptom querying is over, a summary report is created, which can be saved as a pdf (Supplementary Material 1). This summary report includes a summary of (1) present, not present and unsure symptoms, (2) up to five disease suggestions including respective probability, triage advice (e.g., call an ambulance) and symptom importance and (3) basic information about the suggested diseases.

Rheport (www.rheport.de) is an online rheumatology referral system used in Germany to automatically triage appointments of new rheumatology patients according to the respective probability of an inflammatory rheumatic disease (IRD) (9, 10, 18). In contrast to Ada, Rheport is based on a fixed 23-item questionnaire and limited to rheumatic diseases. Furthermore, Rheport does not make any disease suggestions and “only” calculates the individual IRD probability, using an underlying weighted sum score. Based on the IRD probability the patient receives rheumatologist appointment proposals with varying urgency (4 levels). Total scores lower than 1 are transferred (back) to their treating general physician and do not receive rheumatology appointments. Patients with a minimum total score of 1 may book an appointment at a participating rheumatology center and the higher the total score, the earlier the appointment proposals get (total score >4= appointment within 1 week). Once the appointment is accepted, the respective rheumatologist receives a summary report of the questionnaire to guide the appointment (Supplementary Material 2).

### Statistical Analysis

The multicentre prospective design with unequal variances of SUS may lead to heterogeneity of the data and bias the F-value when comparing means. Thus, data were analyzed using robust tests that trim 20% of scores and use a bootstrap procedure to obtain an empirically-derived critical value (*p* < 0.05) against which the test statistic is compared (19–21). The bootstrap procedure was the most effective remedy for non-normality because the critical value was empirically derived from the actual data (21).

The following statistical procedures were used:

- Descriptive statistics including means, standard deviations and quartiles. The percentile bootstrap procedure trimpb, with 2,000 bootstrap samples, was applied to compute robust confidence intervals for trimmed means (21).
- Yuen's test on trimmed means for dependent samples was carried out to calculate difference of SUS. To examine effect size, an explanatory measure of ξ which does not require equal variances and can be generalized to multiple group settings, was used. Values of ξ = 0.10, 0.30, and 0.50 correspond to small, medium, and large effect sizes (22).
- In order to assess within-subject effects (due to repeated measurements) and between-subjects effects (group comparisons), a robust two-way mixed ANOVA using trimmed means was applied by using bwtrim. This method provides a test value (“Q”) which can be used to test null-hypotheses of main effects and interactions (21, 23). In order to conduct the analysis, the age factor was dichotomized using the median value that was detected of the whole sample.

Descriptive statistical analyses were carried out with the software SPSS 22.0. Robust statistical analyses were conducted with the software R 4.1.2 (http://cran.r-project.org) in conjunction with functions of the Wilcox' WRS, WRS2 Robust Statistics package (23).

## Results

### Participant Demographics

**Figure 1:**
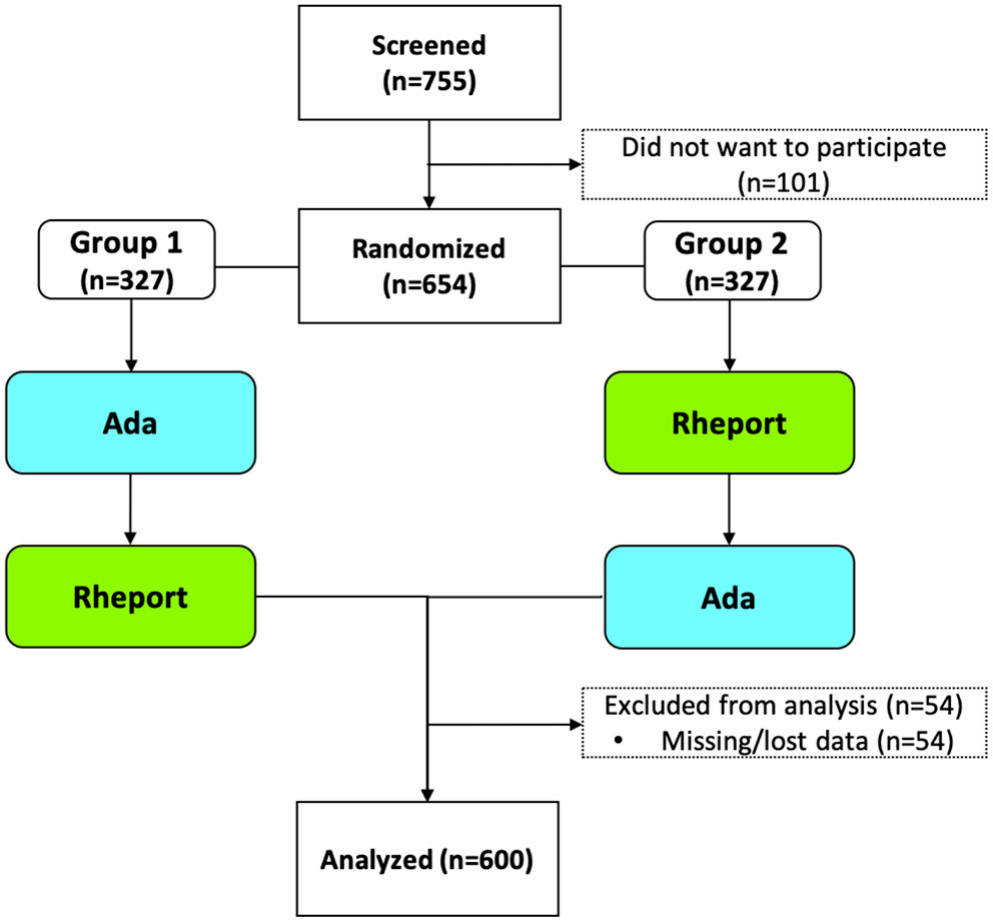
Trial flow chart.

Seven hundred and fifty five consecutive patients newly presenting to three German recruiting rheumatology outpatient clinics with musculoskeletal symptoms and unknown diagnosis were approached between September 2019 and April 2021. Six hundred and fifty four agreed to participate, 54 patients were excluded due to major data missing (one of the DDSS not completed, or questionnaire not started), so that 600 patients were included into the main analysis, see Figure 1. The demographic characteristics are displayed in Table 1. Median age was 52 Years (37.0–61.0), 418/600 (69.7%) patients were female and 214/600 (35.7%) of the patients were diagnosed with an Inflammatory Rheumatic Disease (IRD) based on physician's judgment. 531/600 (88.5%) of the Patients regularly used a smartphone or tablet. 277/600 (46.2%) and 68/600 (11.3%) were using online search engines or dedicated symptom assessment websites/apps previous to their appointment.

**Table 1:** Demographics according to final physician's diagnosis, reported on the discharge summary report.

**Demographics**		**Value**
Age in years, median (IQR)		52 (37.0–61.0)
**Age in years**, ***n*** **(%)**		
18–39		169 (28.2)
40–59		264 (44.0)
>60		167 (27.8)
Sex, female, *n* (%)		418 (69.7)
**Diagnostic categories**, ***n*** **(%)**		
Axial spondyloarthritis		31 (5.2)
Connective tissue disease		22 (3.7)
Crystal arthropathies		8 (1.3)
Peripheral spondyloarthritis		3 (0.5)
Polymyalgia rheumatica		16 (2.7)
Psoriatic arthritis		31 (5.2)
Rheumatoid arthritis		69 (11.5)
Undifferentiated arthritis		19 (3.2)
Vasculitis		8 (1.3)
Other IRDs		7 (1.2)
Osteoarthritis		71 (11.8)
Fibromyalgia		37 (6.2)
Other non-inflammatory		278 (46.3)
**Regular usage**, ***n*** **(%)**		
Smartphone and Tablet		233 (38.8)
Smartphone only		281 (46.8)
Tablet only		17 (2.8)
None		69 (11.5)
**Previous digital symptom assessment**, ***n*** **(%)**		
Online search engines		277 (46.2)
Dedicated symptom assessment websites / apps		68 (11.3)

*IQR, inter-quartile range; IRD, inflammatory rheumatic disease*.

### Symptom Assessment Time and Perceived Helpfulness of Ada, Rheport and Online Search Engines

The median time (IQR) patients spent using online search engines to assess their symptoms was 180 min (120–360 min), compared to Ada with 7 min (6–10 min) and Rheport with 8 min (6–11 min). 111/275 (40.4%) patients rated the online search engine symptom assessment as very helpful or helpful, compared to 266/600 (44.3%) and 395/600 (65.8%) after having used Ada and Rheport, respectively.

### Usability of Ada and Rheport

#### Bivariate Analysis

Patients rated the usability of Rheport significantly higher compared to Ada [79.3/100 vs. 76.7/100 (*p* < 0.0001)], see Table 2. This effect was independent of age and gender (Supplementary Material 3). However, this effect was small, (Supplementary Material 4) and converting the scores to traditional rating categories as previously described by Bangor et al. (16) results in “good” (SUS > 71.4/100 and <85.5/100) and above average (SUS > 68) (14) usability for both SUS. Older patients (>52 years) rated DDSS usability significantly (*p* < 0.0001) lower compared to younger patients (≤52 years), see (Supplementary Material 3). The effect of age was medium or close to medium with values of 0.35 and 0.29 for Rheport and Ada, respectively.

**Table 2:** Usability ratings of Ada and Rheport using the System Usability Scales (SUS).

	**Ada**		**Rheport**			
**SUS Item**	**Mean (SD)**	**Mean trimmed (95% CI)**	**Mean (SD)**	**Mean trimmed (95% CI)**	**ξ^a^**	***P*-Value^b^**
1-I think I would like to use the system frequently	1.9 (1.3)	2.0 (1.8–2.1)	2.1 (1.2)	2.2 (2.1–2.3)	**0.10**	**0.0004**
2-I found the system to be unnecessarily complex	3.1 (1.1)	3.3 (3.2–3.5)	3.2 (1.0)	3.4 (3.3–3.6)	0.05	0.0749
3-I thought the system was easy to use	3.2 (1.1)	3.6 (3.6–3.7)	3.3 (1.1)	3.7 (3.6–3.7)	0.04	0.1740
4-I think that I would need support of a technical person to be able to use the system	3.3 (1.2)	3.8 (3.8–3.9)	3.4 (1.1)	3.9 (3.8–3.9)	0.03	0.1581
5-I found the various functions in the system were well integrated	2.6 (1.1)	2.7 (2.6–2.8)	2.9 (1.0)	2.9 (2.8–3.0)	**0.11**	**0.0003**
6-I thought there was too much inconsistency in the system	2.6 (1.0)	2.5 (2.4–2.6)	2.7 (1.0)	2.7 (2.6–2.8)	0.09	**0.0009**
7-I would imagine that most people would learn to use the system very quickly	3.1 (1.1)	3.3 (3.2–3.4)	3.1 (1.1)	3.4 (3.3–3.6)	0.06	**0.0220**
8-I found the system very cumbersome to use	3.4 (1.0)	3.7 (3.7–3.8)	3.4 (0.9)	3.8 (3.7–3.8)	0.03	0.3623
9-I felt very confident using the system	3.0 (1.1)	3.3 (3.2–3.4)	3.2 (1.1)	3.5 (3.3–3.6)	**0.10**	**0.0003**
10-I needed to learn a lot of things before I could get going with the system	3.5 (1.0)	3.9 (3.9–4.0)	3.6 (0.9)	4.0 (3.9–4.0)	0.01	0.7859
Total Score (100)	74.4 (16.8)	76.7 (75.3–78.2)	77.1 (16.0)	79.3 (78.0–80.6)	**0.11**	**0.0000**

a *ξ, explanatory measure of effect size*;

b *Yuen's test on trimmed means for dependent samples. Bold values indicate the at least small effect size and statistically significant*.

#### Multivariate Analysis

A robust two-way mixed ANOVA of usability (SUS) for the whole sample with DDSS and age as factors confirmed the effect of DDSS (Supplementary Material 4). Rheport was rated with a higher total SUS score (Q = 22.7; *p* < 0.0001). In this analysis, we confirmed the significant effect of age (Q = 32.7; *p* < 0.0001) resulting in lower usability rating for older patients. There was not any significant interaction between effects of age and DDSS (Q = 1.1; *p* = 0.31).

However, if the analysis was conducted separately for each gender, the results were different. In the male subsample, the effects of DDSS (Q = 5.4; *p* = 0.02) and age (Q = 8.3; *p* = 0.005) as well as age by DDSS interactions (Q = 1.1; *p* = 0.31) were comparable to those described for the whole sample. In the female subsample, the effects of DDSS and age were both highly significant (Q = 14.7; *p* = 0.0002, Q = 23.8; *p* < 0.0001, respectively), but the effect of DDSS on usability ratings was different for younger patients than it was for older participants (Q = 7.9; *p* = 0.005), suggesting that younger women (<52 years) showed greater usability rating differences between Ada and Rheport.

### Acceptability of Ada and Rheport

440/600 (73.3%) and 475/600 (79.2%) of the patients would recommend Ada and Rheport to friends and other patients, respectively. For both DDSS, a higher proportion of older patients (≥55 years) compared to younger patients (≥18–39 years) recommended the DDSS to friends and other patients (Table 3).

**Table 3:** Usage of online assessment tools prior to visit and acceptance of DDSS according to respective age groups and in comparison to previous studies.

**Age group**	**Used online search engines previous to appointment (*N* = 600)**	**Used dedicated symptom assessment website/app previous to appointment (*N* = 600)**	**Healthwatch Enfield (24); “would use a symptom checker before seeking advice from GP” (*N* = 1,071)^**a**^**	**Miller et al. (12); “Extremely Likely/Likely to recommend Ada to a friend or relative” (*N* = 447)**	**would recommend Ada to a friend or other patient (*N* = 600)**	**would recommend Rheport to a friend or other patient (*N* = 600)**
18–24, *n*/*N* (%)	23/36 (63.9)	4/36 (11.1)	(74)	50/54 (92.6)	24/36 (66.7)	29/36 (80.6)
25–39 *n/N* (%)	78/133 (58.6)	17/133 (12.8)	(71)	125/147 (85.0)	95/133 (71.4)	98/133 (73.7)
40–54, *n/N* (%)	90/183 (49.2)	28/183 (15.3)	(69)	121/141 (85.8)	134/183 (73.2)	146/183 (79.8)
55–69, *n/N* (%)	82/194 (42.3)	15/194 (7.7)	(51)	64/72 (88.9)	144/194 (74.2)	156/194 (80.4)
70+, *n/N* (%)	4/54 (7.4)	4/54 (7.4)	(34)	25/33 (75.8)	43/54 (79.6)	46/54 (85.2)

a *n/N values are missing and only percentage is reported*.

### Potential Impact of Ada and Rheport

482/600 (80.3%) and 506/600 (84.3%) of the patients declared that they would not have done anything differently after having used Ada and Rheport, respectively, see Table 4. 68/600 (11.3%) and 61/600 (10.2%) stated that they would have consulted a physician earlier, whereas 7/600 (1.2%) and 6/600 (1.0%) stated that they would have consulted a physician later, having used Ada and Rheport, respectively. 17/600 (2.8%) and 14/600 (2.3%) would have worried less and 12/600 (2.0%) and 7/600 (1.2%) would have worried more after having used Ada and Rheport, respectively. 526/600 (87.7%) patients would like to be able to choose an appointment directly from a list of qualified doctors in the surrounding area at the end of the symptom assessment.

**Table 4:** Self-reported potential effects of diagnostic decision support system assessments^a^.

**Would you have done anything different having used the DDSS?** ***n*** **(%)**		
	**Ada**	**Rheport**
No, nothing	482 (80.3)	506 (84.3)
Yes, seek a physician appointment earlier	68 (11.3)	61 (10.2)
Yes, seek a physician appointment later	7 (1.2)	6 (1.0)
Yes, seek no physician appointment at all	0 (0.0)	0 (0.0)
Yes, seek an appointment with a physician with a different specialty	11 (1.8)	5 (0.8)
Yes, worry less	17 (2.8)	14 (2.3)
Yes, worry more	12 (2.0)	7 (1.2)
*Missing values*	*3 (0.5)*	*1 (0.2)*

a *Answers were not mutually exclusive*.

## Discussion

To our knowledge this is the first and largest study directly comparing the patient perceived acceptability, usability and usage time of two digital symptom assessment systems including an artificial intelligence and chatbot-based symptom checker (Ada) and an online questionnaire-based self-referral tool (Rheport). Additionally, patient perceived helpfulness and usage time of these two DDSS was put in perspective, by comparison to previous conventional online search engine usage (if performed by patients previous to the appointment) and the potential impact of DDSS usage was investigated.

In line with previous studies (2, 3, 6, 7), our study showed that a significant proportion of patients consulting rheumatology services assessed their symptoms online prior to their appointment (46%). The proportion of patients using online search engines prior to their visit decreased with age (Table 3). One reason for this might be the decreasing eHealth literacy with age (3), with older patients not feeling confident looking for health-related information online.

In contrast to online search engines, only a minority used dedicated symptom assessment websites/apps (46 vs. 11%). In general, patients spent more time assessing symptoms online using search engines compared to the usage time of both DDSS (180 vs. 7/8 min). On the other hand, more patients stated that DDSS usage was helpful/very helpful compared to using search engines (44/66 vs. 40%), suggesting that using DDSS instead of conventional online search engines could save time and increase helpfulness.

In a recent online survey study Kernder et al. (2) could show that the COVID pandemic lead to an increased usage of symptom checkers by rheumatic patients and rheumatologists. 40.5% (121/299) of rheumatic patients stated that they already used a symptom checker, however this larger proportion of patients (40.5 vs. 11.3%) is probably due to the selection bias due to the online nature of survey study, suggesting that digitally active patients are more likely to use DDSS. In line with this, our study shows that with increasing age the proportion of patients having used online search engines or dedicated symptom assessment website/app previous to their appointment is steadily declining (Table 3).

Overall, 79 vs. 73% of the patients would recommend Rheport and Ada to friends and other patients, respectively. The preference for Rheport compared to Ada was largest in the youngest age group (80 vs. 67%) and particularly in female patients. Furthermore, we observed a higher symptom checker recommendation rate in older patients compared to younger patients, in contrast to previous studies (12, 24) (Table 3). Similarly, the usability (SUS) of questionnaire-based Rheport was rated significantly higher compared to AI-based Ada (77.1/100 vs. 74.4/100). However, the effect was weak (“small effect” with values from 0.10 to 0.29 in the bivariate analysis), which can be attributed to the large sample size. Translating these numeric results to categories, the usability of both DDSS was “good.”

Similar to a previous observational study analyzing the potential impact of Ada (12), the majority of patients (already being at the healthcare facility) declared that they would not have done anything differently after having used the DDSS. In line with previous work that showed a general risk adversity of symptom checkers (4, 11), our results shows that DDSS suggestions would actually encourage patients to seek earlier care rather than turn to self-care or see a physician later. On the other hand, Meyer et al. (13) reported that 14/26 (54%) of symptom checkers users given advice to proceed to the ED actually did.

The large majority of patients would welcome the option to book an appointment directly from a list of qualified doctors in the surrounding area at the end of the symptom assessment. This feature is already implemented in Rheport. The AI-based Isabel symptom checker, for example also offers users to contact a doctor and “find a lab test” after symptom assessment (13).

A strength of this study is the large sample of patients, usage of a validated instrument to measure usability and the study's real-world nature. Furthermore, the fact that the same patient provided feedback on two DDSS and their previous online symptom assessment is a major strength of the study. The study also has several limitations. Although this study included three centers, the findings are limited to one country and patients referred to rheumatology services. Since the robust mixed ANOVA is actually limited to two-way design, the gender factor was not included in the calculation and the analysis was conducted separately for the male and female subsamples. We did not measure in how many cases patients needed help to use the DDSS, and we only asked patients about the “theoretical” impact of using the DDSS. As patients often used online search engines months before the appointment, this data is subject to recall bias and should be interpreted carefully. Furthermore, we did not differentiate between various available online search engines. No separate power calculation was carried out for the evaluation of the secondary outcomes investigated in this study. The risk-adverse setting, “where patients continue to receive standard care” was intentionally chosen as recommended by Fraser et al. (25). Furthermore, we did not assess the (e)health literacy of participants.

This study suggests that the majority of patients find both DDSS helpful and easy to use. From a physician perspective, the possibility to obtain a structured summary of the patient medical history, ready to guide the appointment and to be imported into the electronic health record appeals time saving and helpful as well. However, due to Ada's variable questioning approach, determining new questions on all previously supplied basic health information, Ada bears the risk of leaving out important questions compared to a fixed-questionnaire approach (Rheport). Whereas, Rheport uses pictures of typical symptoms (swollen joints), Ada does not. Pictures might aid to specify definitions to reduce interpretation differences of symptoms (26). Although we did not specifically ask patients about this difference, that could have at least partially have contributed to the better perception of Rheport by patients. Future qualitative research could complement the present findings by adding detailed reasons for the observed rating differences. Furthermore, a physician-based study could evaluate the time-saving potential and perceived helpfulness of DDSS.

## Conclusion

Patients increasingly assess their symptoms independently online, however only a minority used dedicated symptom assessment websites or diagnostic decision support systems. DDSS are easy to use, well accepted among patients with musculoskeletal complaints and could replace online search engines for patient symptom assessment saving time and increasing helpfulness.

## Data Availability Statement

The raw data supporting the conclusions of this article will be made available by the authors, without undue reservation.

## Ethics Statement

The studies involving human participants were reviewed and approved by the Ethics Committee of the Medical Faculty of the University of Erlangen-Nürnberg, Germany. The patients/participants provided their written informed consent to participate in this study.

## Author Contributions

JK, AH, GS, PB-B, WV, and MW: conceptualization. JK, DS, AK, SB, CB, and HL: methodology. FFu, JM, WV, MW, PB-B, and JK: software. JK, FFu, JM, FM, HM, YI, NV, MW, and AH: formal analysis. JK, FFu, HM, and JM: data curation. JK and FM: writing—original draft preparation. JK, FM, FFa, NV, YI, AH, and MW: writing—review and editing. JK: visualization. GS, AR, and JD: supervision. JK, MW, and AH: funding acquisition. All authors have read and agreed to the published version of the manuscript.

## Funding

This study was supported by Novartis Pharma GmbH, Nürnberg, Germany (Grant Number: 33419272). The APC was funded by Novartis Pharma GmbH, Nürnberg, Germany (Grant Number: 33419272). Novartis employees were not involved in the design and conduct of the study. They did not contribute to the collection, analysis, and interpretation of data. They did not support the authors in the development of the manuscript.

## Conflict of Interest

JK and has received research support from Novartis Pharma GmbH. Qinum and RheumaDatenRhePort developed and hold rights for Rheport. WV, PB-B, and MW are members of RheumaDatenRhePort. WV and PB-B were involved in the development of Rheport. JK is a member of the scientific board of RheumaDatenRhePort. The remaining authors declare that the research was conducted in the absence of any commercial or financial relationships that could be construed as a potential conflict of interest.

## Publisher's Note

All claims expressed in this article are solely those of the authors and do not necessarily represent those of their affiliated organizations, or those of the publisher, the editors and the reviewers. Any product that may be evaluated in this article, or claim that may be made by its manufacturer, is not guaranteed or endorsed by the publisher.

## References

[1] Wyatt JC. Fifty million people use computerised self triage. BMJ. (2015) 351:h3727. 10.1136/bmj.h3727 26156750

[2] Kernder A Morf H Klemm P Vossen D Haase I Mucke J. Digital rheumatology in the era of COVID-19: results of a national patient and physician survey. RMD Open. (2021) 7:e001548. 10.1136/rmdopen-2020-001548 33622673 PMC7907631

[3] Knitza J Simon D Lambrecht A Raab C Tascilar K Hagen M. Mobile health usage, preferences, barriers, and ehealth literacy in rheumatology: patient survey study. JMIR Mhealth Uhealth. (2020) 8:e19661. 10.2196/19661 32678796 PMC7450373

[4] Semigran HL Linder JA Gidengil C Mehrotra A. Evaluation of symptom checkers for self diagnosis and triage: audit study. BMJ. (2015) 351:h3480. 10.1136/bmj.h3480 26157077 PMC4496786

[5] Knitza J Krusche M Leipe J. [Digital diagnostic support in rheumatology]. Z Rheumatol. (2021) 80(10):909–13. 10.1007/s00393-021-01097-x 34605980

[6] Proft F Spiller L Redeker I Protopopov M Rodriguez VR Muche B. Comparison of an online self-referral tool with a physician-based referral strategy for early recognition of patients with a high probability of axial spa. Semin Arthritis Rheum. (2020) 50:1015–21. 10.1016/j.semarthrit.2020.07.018 32911279

[7] Powley L McIlroy G Simons G Raza K. Are online symptoms checkers useful for patients with inflammatory arthritis? BMC Musculoskelet Disord. (2016) 17:362. 10.1186/s12891-016-1189-2 27553253 PMC4995741

[8] Gilbert S Mehl A Baluch A Cawley C Challiner J Fraser H. How accurate are digital symptom assessment apps for suggesting conditions and urgency advice? a clinical vignettes comparison to GPs. BMJ Open. (2020) 10:e040269. 10.1136/bmjopen-2020-040269 33328258 PMC7745523

[9] Kleinert S Bartz-Bazzanella P von der Decken C Knitza J Witte T Fekete SP. A real-world rheumatology registry and research consortium: the german rheumadatenrheport (RHADAR) registry. J Med Internet Res. (2021) 23:e28164. 10.2196/preprints.28164 34014170 PMC8176344

[10] Knitza J Mohn J Bergmann C Kampylafka E Hagen M Bohr D. Accuracy, patient-perceived usability, and acceptance of two symptom checkers (Ada and Rheport) in rheumatology: interim results from a randomized controlled crossover trial. Arthritis Res Ther. (2021) 23:112. 10.1186/s13075-021-02498-8 33849654 PMC8042673

[11] Schmieding ML Mörgeli R Schmieding MAL Feufel MA Balzer F. Benchmarking triage capability of symptom checkers against that of medical laypersons: survey study. J Med Internet Res. (2021) 23:e24475. 10.2196/24475 33688845 PMC7991983

[12] Miller S Gilbert S Virani V Wicks P. Patients' utilization and perception of an artificial intelligence-based symptom assessment and advice technology in a british primary care waiting room: exploratory pilot study. JMIR Hum Factors. (2020) 7:e19713. 10.2196/19713 32540836 PMC7382011

[13] Meyer AND Giardina TD Spitzmueller C Shahid U Scott TMT Singh H. Patient perspectives on the usefulness of an artificial intelligence–assisted symptom checker: cross-sectional survey study. J Med Internet Res. (2020) 22:e14679. 10.2196/14679 32012052 PMC7055765

[14] Bangor A Kortum PT Miller JT. An empirical evaluation of the system usability scale. Int J Hum Comput Int. (2008) 24:574–94. 10.1080/10447310802205776

[15] Gronier G Baudet A. Psychometric evaluation of the F-SUS: creation and validation of the french version of the system usability scale. Int J Hum Comput Int. (2021) 37:1571–82. 10.1080/10447318.2021.1898828

[16] Bangor A Kortum P Miller J. Determining what individual SUS scores mean: adding an adjective rating scale. J Usability Studies. (2009) 4:114–23. 10.5555/2835587.2835589

[17] Butcher M. Ada Health Built An Ai-Driven Startup By Moving Slowly And Not Breaking Things. TechCrunch. Available online at: https://social.techcrunch.com/2020/03/05/move-slow-and-dont-break-things-how-to-build-an-ai-driven-startup/ (accessed January 29, 2021).

[18] Benesova K Lorenz H-M Lion V Voigt A Krause A Sander O. [Early recognition and screening consultation: a necessary way to improve early detection and treatment in rheumatology? : overview of the early recognition and screening consultation models for rheumatic and musculoskeletal diseases in Germany]. Z Rheumatol. (2019) 78:722–42. 10.1007/s00393-019-0683-y 31468170

[19] Field AP Wilcox RR. Robust statistical methods: a primer for clinical psychology and experimental psychopathology researchers. Behav Res Ther. (2017) 98:19–38. 10.1016/j.brat.2017.05.013 28577757

[20] Wilcox RR Keselman HJ. Modern robust data analysis methods: measures of central tendency. Psychol Methods. (2003) 8:254–74. 10.1037/1082-989X.8.3.254 14596490

[21] Wilcox Rand. Introduction to Robust Estimation and Hypothesis Testing. 4th ed. Elsevier (2016). 10.1016/B978-0-12-804733-0.00001-9

[22] Wilcox RR Tian TS. Measuring effect size: a robust heteroscedastic approach for two or more groups. J Appl Stat. (2011) 38:1359–68. 10.1080/02664763.2010.498507

[23] Mair P Wilcox R. Robust statistical methods in R using the WRS2 package. Behav Res Methods. (2020) 52:464–88. 10.3758/s13428-019-01246-w 31152384

[24] Healthwatch Enfield. Using Technology to Ease The Burden on Primary Care (2019). Available online at: https://healthwatchenfield.co.uk/~wp-content/uploads/2019/01/Report_UsingTechnologyToEaseTheBurdenOnPrimaryCare.pdf

[25] Fraser H Coiera E Wong D. Safety of patient-facing digital symptom checkers. Lancet. (2018) 392:2263–4. 10.1016/S0140-6736(18)32819-8 30413281

[26] Moorman PW Siersema PD de Ridder MA van Ginneken AM. How often is large smaller than small? Lancet. (1995) 345:865. 10.1016/S0140-6736(95)93004-3 7898256

