# Supplementary material for: Patient's Perception of Digital Symptom Assessment Technologies in Rheumatology: Results From a Multicentre Study
Source: Front Public Health. 2022 Feb 22;10:844669. doi: 10.3389/fpubh.2022.844669 (PMC8902046; doi:10.3389/fpubh.2022.844669)
Supplement: Supplementary file 1 [file Data_Sheet_1.docx]

**Supplementary Material 1.** Example of the Ada summary report

**
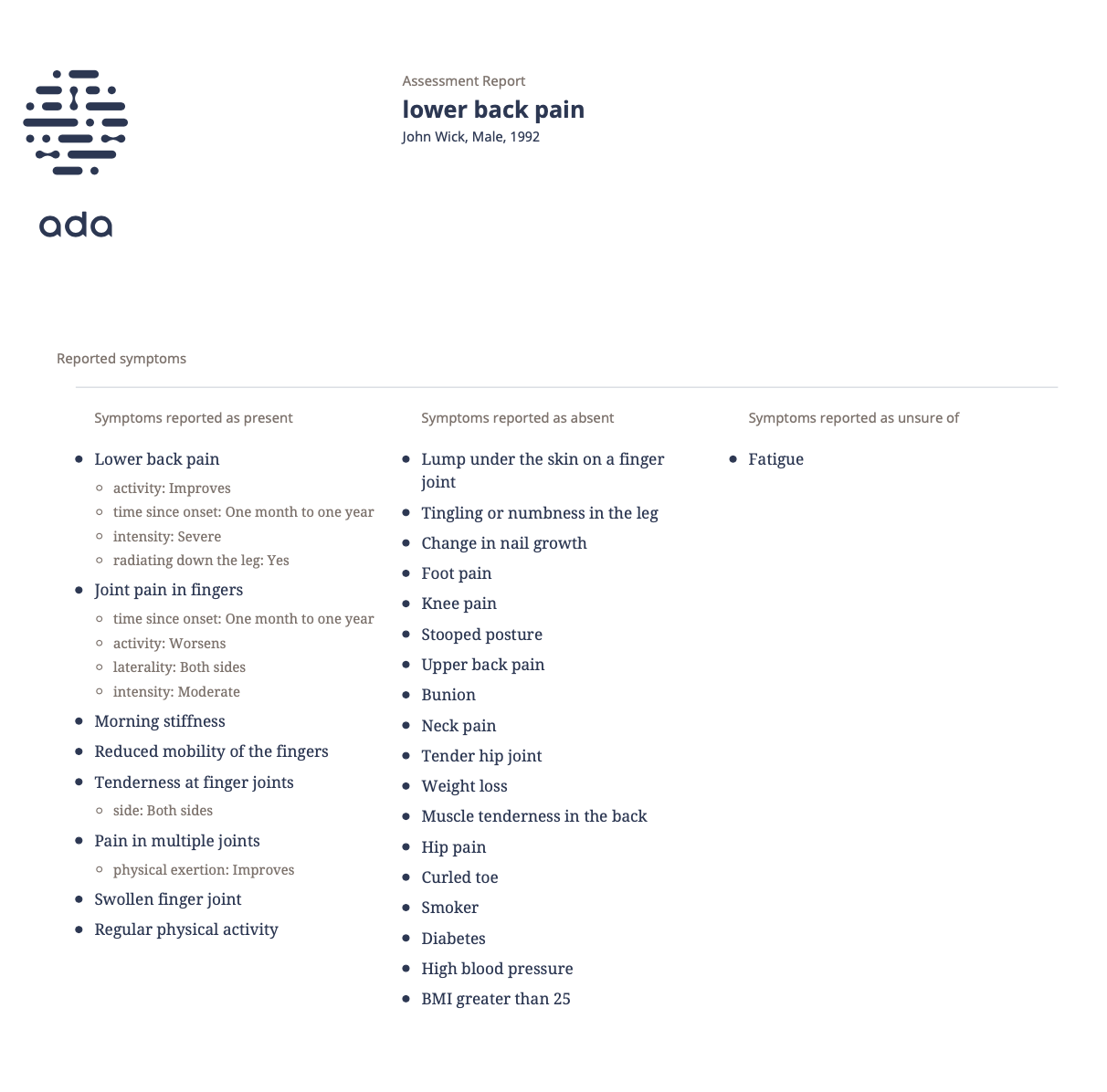
**

**
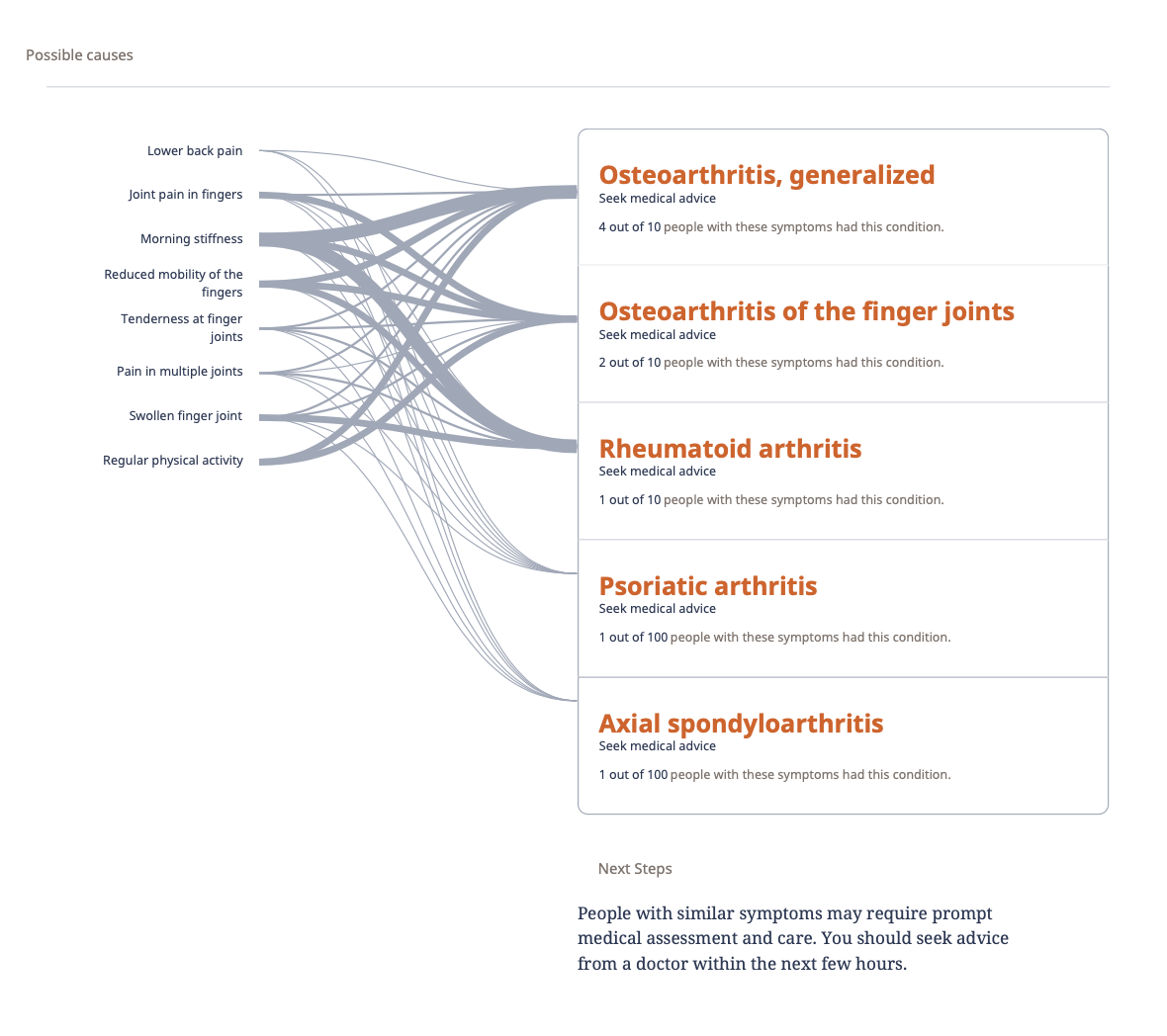
**

**
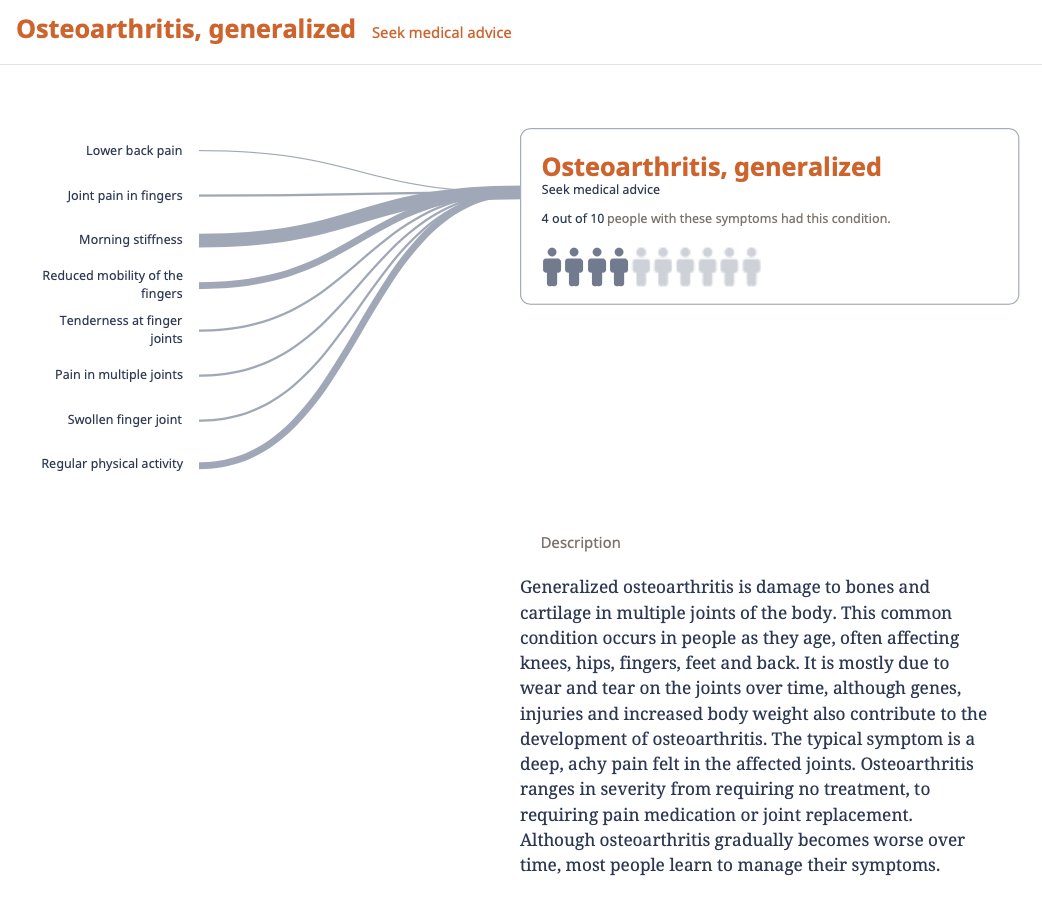
**

**
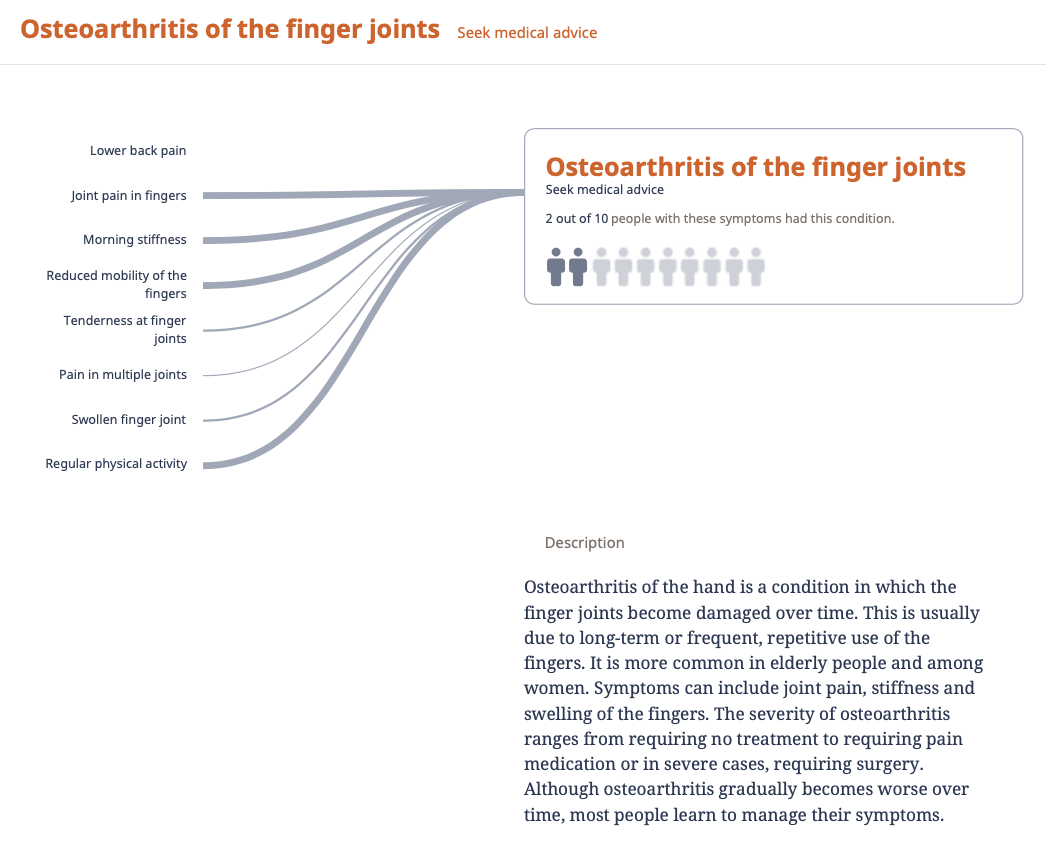
**

**
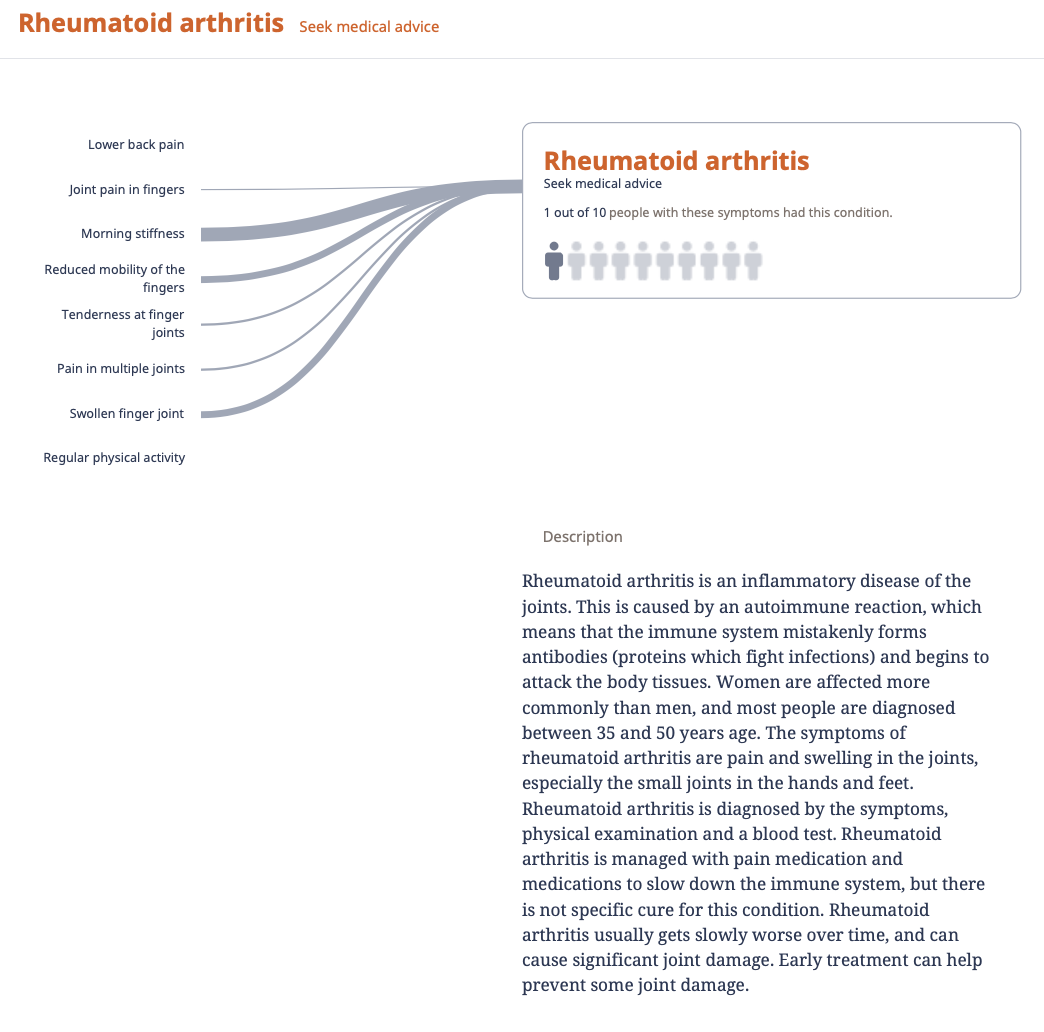

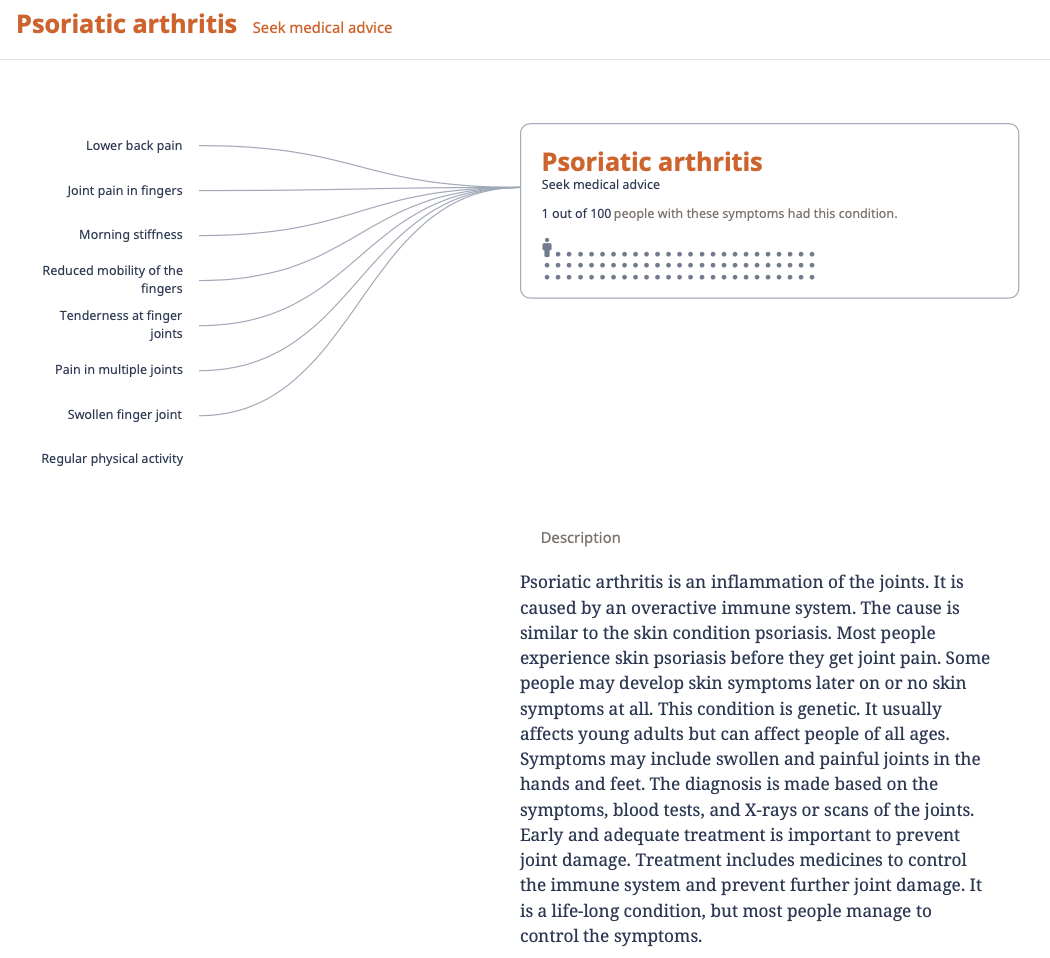

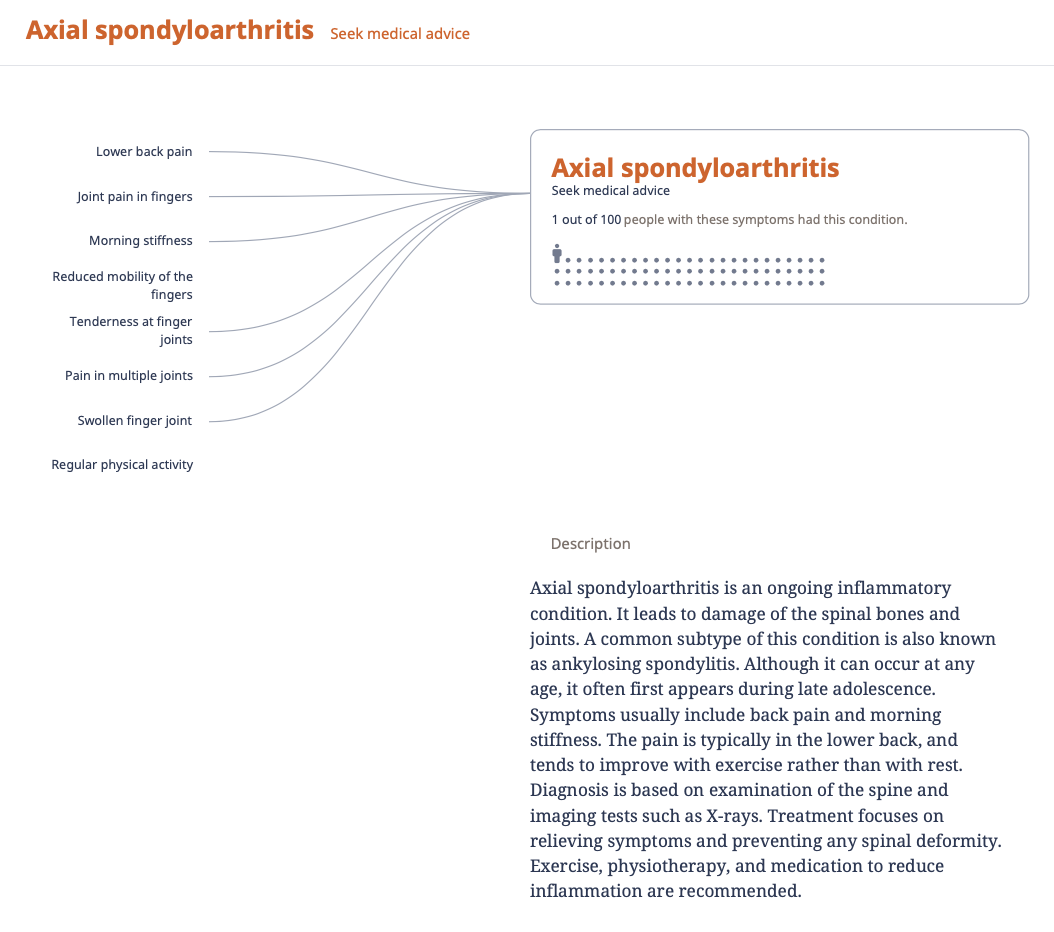
**

**Supplementary Material 2.** Example of the Rheport questionnaire report.

| **Question** | **Answers** | **Factor** | **Weight** | **Sub-score** |
| --- | --- | --- | --- | --- |
| Gender | Female | 0 | 0 % | 0.00 |
| Age | < 60 years | 0 | 2 % | 0.00 |
| Weight loss | No | 0 | 3 % | 0.00 |
| Duration of complaints | 6 months, < 12 months | 3 | 5 % | 0.15 |
| Preceding injury | No | 5 | 1 % | 0.05 |
| Preceding infection | No | 0 | 1 % | 0.00 |
| Preceding tick sting | No | 0 | 0 % | 0.00 |
| Referring physician | General practitioner | 0 | 1 % | 0.00 |
| Lab results | No lab results available | 0 | 10 % | 0.00 |
| Family history | No | 0 | 0 % | 0.00 |
| Joint pain | With movement | 1 | 10 % | 0.10 |
| Joint swelling | Big toe | 10 | 10 % | 1.00 |
| Finger swelling | Whole finger | 10 | 4.5 % | 0.45 |
| Duration of joint swelling | > 6 weeks, < half a year | 10 | 2.5 % | 0.25 |
| Joint stiffness | All day long | 1 | 7.5 % | 0.075 |
| Headache | Neck and back of head | 1 | 7.5 % | 0.075 |
| Lower back pain | No | 0 | 7.5 % | 0.00 |
| Other pain | No | 0 | 7.5 % | 0.00 |
| Pain related limitation of movement | No | 0 | 2.5 % | 0.00 |
| Muscle weakness | No | 0 | 10 % | 0.00 |
| General symptoms | Disrupted sleep, often tired | 0 | 2.5 % | 0.00 |
| Other symptoms | Fever > 38°C | 4 | 2.5 % | 0.10 |
| Comorbidities | Psoriasis | 4 | 2.5 % | 0.10 |
|  | **Total score** | |  | **2.35** |

**Supplementary Material 3.** Effects of age and digital applications on System Usability Scale (SUS) scores according to gender.

| **Women** | | | | | | | | | | | | |
| --- | --- | --- | --- | --- | --- | --- | --- | --- | --- | --- | --- | --- |
|  | **ADA** | | | | | **Rheport** | | | | | **ξ ^a^** | **P-Value ^b^** |
| **SUS Item ^c^** | **Q25** | **Q50** | **Q75 ^d^** | **Mean (SD) ^e^** | **Mean tr (95% CI) ^f^** | **Q25** | **Q50** | **Q75** | **Mean (SD)** | **Mean tr (95% CI)** |  |  |
| **1** | 1 | 2 | 3 | 1.9 (1.3) | 2.0 (1.9-2.1) | 1 | 2 | 3 | 2.2 (1.3) | 2.2 (2.1-2.3) | **0.10** | **.0031** |
| **2** | 2 | 4 | 4 | 3.1 (1.1) | 3.4 (3.2-3.5) | 2 | 4 | 4 | 3.2 (1.0) | 3.5 (3.3-3.6) | 0.04 | .2066 |
| **3** | 3 | 4 | 4 | 3.3 (1.1) | 3.6 (3.5-3.7) | 3 | 4 | 4 | 3.3 (1.1) | 3.7 (3.6-3.8) | **0.07** | **.0493** |
| **4** | 3 | 4 | 4 | 3.3 (1.2) | 3.8 (3.7-3.9) | 3 | 4 | 4 | 3.4 (1.1) | 3.9 (3.8-3.9) | 0.05 | .0693 |
| **5** | 2 | 3 | 4 | 2.7 (1.1) | 2.8 (2.6-2.9) | 2 | 3 | 4 | 2.9 (1.0) | 2.9 (2.8-3.1) | **0.10** | **.0029** |
| **6** | 2 | 2 | 3 | 2.6 (1.0) | 2.6 (2.4-2.7) | 2 | 3 | 4 | 2.8 (1.0) | 2.7 (2.6-2.9) | **0.10** | **.0007** |
| **7** | 3 | 3 | 4 | 3.1 (1.1) | 3.4 (3.2-3.5) | 3 | 3.5 | 4 | 3.2 (1.0) | 3.5 (3.4-3.6) | **0.07** | **.0024** |
| **8** | 3 | 4 | 4 | 3.5 (0.9) | 3.8 (3.7-3.9) | 3 | 4 | 4 | 3.5 (0.9) | 3.8 (3.7-3.9) | **0.01** | **.0079** |
| **9** | 2 | 3 | 4 | 3.0 (1.1) | 3.3 (3.2-3.5) | 3 | 4 | 4 | 3.2 (1.0) | 3.5 (3.4-3.6) | **0.10** | **.0023** |
| **10** | 4 | 4 | 4 | 3.5 (1.0) | 4.0 (4.0-4.0) | 4 | 4 | 4 | 3.6 (0.9) | 3.9 (4.0-4.0) | 0.00 | 1.000 |
| **Total Score** | 65 | 77.5 | 87.5 | 75.3 (16.2) | 77.5 (75.8-79.3) | 67.5 | 80 | 90 | 78.0 (15.7) | 80.1 (78.6-81.7) | **0.11** | **.0000** |
| **Men** | | | | | | | | | | | | |
| **1** | 1 | 2 | 3 | 1.8 (1.2) | 1.9 (1.7-2.0) | 1 | 2 | 3 | 2.0 (1.2) | 2.0 (1.8-2.2) | 0.10 | .0510 |
| **2** | 2 | 3 | 4 | 3.0 (1.1) | 3.3 (3.1-3.5) | 3 | 3 | 4 | 3.2 (0.9) | 3.4 (3.2-3.6) | 0.07 | .1846 |
| **3** | 3 | 4 | 4 | 3.1 (1.2) | 3.5 (3.3-3.7) | 3 | 4 | 4 | 3.1 (1.1) | 3.5 (3.2-3.7) | 0.03 | .6543 |
| **4** | 3 | 4 | 4 | 3.3 (1.2) | 3.8 (3.7-3.9) | 3 | 4 | 4 | 3.4 (1.1) | 3.8 (3.7-7.9) | 0.01 | .8284 |
| **5** | 2 | 3 | 3 | 2.5 (1.1) | 2.6 (2.5-2.7) | 2 | 3 | 4 | 2.8 (1.0) | 2.8 (2.6-3.0) | **0.12** | **.0386** |
| **6** | 2 | 2 | 3 | 2.5 (1.0) | 2.4 (2.3-2.5) | 2 | 2 | 3 | 2.6 (1.0) | 2.5 (2.3-2.7) | 0.05 | .3078 |
| **7** | 2 | 3 | 4 | 3.0 (1.1) | 3.2 (3.0-3.4) | 2 | 3 | 4 | 3.0 (1.1) | 3.2 (3.0-3.4) | 0.01 | .7494 |
| **8** | 3 | 4 | 4 | 3.3 (0.9) | 3.6 (3.5-3.7) | 3 | 4 | 4 | 3.3 (1.0) | 3.7 (3.4-3.9) | 0.08 | .3076 |
| **9** | 2 | 3 | 4 | 2.9 (1.1) | 3.2 (3.0-3.4) | 3 | 3 | 4 | 3.1 (1.1) | 3.4 (3.1-3.6) | **0.10** | **.0466** |
| **10** | 3.3 | 4 | 4 | 3.5 (1.0) | 3.9 (3.8-4.0) | 4 | 4 | 4 | 3.6 (0.9) | 3.9 (3.8-4.0) | 0.02 | .6194 |
| **Total Score** | 60 | 77.5 | 85.0 | 72.3 (17.9) | 74.7 (71.8-77.6) | 65 | 77.5 | 100 | 74.9 (16.6) | 77.1 (74.4-80.0) | **0.10** | **.0312** |

^a^ξ = explanatory measure of effect size; ^b^ Yuen’s test on trimmed means for dependent samples; ^c^ transformed SUS items; ^d^Q25, Q50, Q75 = 25th quantile, Median, 75th quantile; ^e^Mean (SD) = mean with standard deviation; ^f^Mean tr = trimmed mean with 95% confidence interval.

**Supplementary Material 4.** Effects of age and Diagnostic Decision Support System (DDSS) on System Usability Scale (SUS) scores according to gender.

| **Effect** | **System Usability Scale Items (Item, Total Score)** | | | | | | | | | | | | | | | | | | | | | |
| --- | --- | --- | --- | --- | --- | --- | --- | --- | --- | --- | --- | --- | --- | --- | --- | --- | --- | --- | --- | --- | --- | --- |
|  | **1** | | **2** | | **3** | | **4** | | **5** | | **6** | | **7** | | **8** | | **9** | | **10** | | **Total Score** | |
|  | **Q^b^** | **P ^c^** | **Q** | **P** | **Q** | **P** | **Q** | **P** | **Q** | **P** | **Q** | **P** | **Q** | **P** | **Q** | **P** | **Q** | **P** | **Q** | **P** | **Q** | **P** |
| **Women (N=418)** | | | | | | | | | | | | | | | | | | | | | | |
| **Age** | 1.3 | .2518 | 8.7 | .**0034** | 16.4 | **.0001** | 31.5 | **.0000** | 1.0 | .3298 | 4.2 | **.0419** | 1.4 | .2454 | 7.5 | **.0066** | 14.4 | **.0002** | 14.6 | **.0002** | 23.8 | **.0000** |
| **DDSS** | 8.0 | **.0050** | 1.7 | .1904 | 1.6 | .2070 | 1.4 | .2411 | 9.0 | **.0030** | 11.7 | **.0007** | 2.7 | .1022 | 0.2 | .6617 | 5.8 | **.0168** | 0.1 | .8097 | 14.7 | **.0002** |
| **Age X DDSS^d^** | 1.4 | .2391 | 0.7 | .4171 | 7.2 | **.0077** | 1.4 | .2411 | 0.6 | .4541 | 1.6 | .2012 | 2.2 | .1354 | 6.5 | **.0118** | 0.1 | .7483 | 0.1 | .8097 | 7.9 | **.0053** |
| **Men (N=182)** | | | | | | | | | | | | | | | | | | | | | | |
| **Age** | 0.3 | .5944 | 4.2 | **.0436** | 7.7 | **.0067** | 13.4 | **.0005** | 4.3 | **.0406** | 0.7 | .3995 | 1.5 | .2250 | 2.0 | .1565 | 7.6 | **.0067** | 6.8 | **.0119** | 8.3 | **.0048** |
| **DDSS** | 3.9 | .0521 | 1.8 | .1791 | 0.4 | .5405 | 0.4 | .5246 | 4.6 | **.0337** | 0.3 | .5965 | 0.1 | .7112 | 0.8 | .3638 | 4.1 | **.0461** | 0.8 | .3735 | 5.4 | **.0218** |
| **Age X DDSS** | 0.3 | .6009 | 0.2 | .6679 | 0.4 | .5405 | 1.4 | .2279 | 2.0 | .1584 | 0.8 | .3617 | 2.0 | .1630 | 0.2 | .6296 | 0.2 | .6986 | 0.8 | .3735 | 1.1 | .3077 |
| **Total Sample (N=600)** | | | | | | | | | | | | | | | | | | | | | | |
| **Age** | 0.5 | .4831 | 13.9 | **.0002** | 28.2 | **.0000** | 47.2 | **.0000** | 1.9 | .1659 | 4.2 | **.0410** | 5.4 | **.0209** | 11.5 | **.0008** | 24.3 | **.0000** | 22.3 | **.0000** | 32.7 | **.0000** |
| **DDSS** | 12.7 | **.0004** | 3.2 | .0729 | 0.4 | .5279 | 2.4 | .1250 | 13.1 | **.0003** | 11.2 | **.0009** | 4.2 | **.0421** | 0 | .8252 | 11.4 | **.0008** | 0.7 | .4124 | 22.7 | **.0000** |
| **Age X DDSS** | 0.4 | .5183 | 0.1 | .7383 | 3.4 | .0646 | 2.4 | .1250 | 1.0 | .3200 | 0 | .8425 | 0.3 | .6059 | 9.3 | **.0025** | 0.5 | .4598 | 0.7 | .4124 | 1.1 | .3053 |

^a^Robust ANOVA (mixed design), ^b^Q=Robust ANOVA test statistic for trimmed means, ^C^P-Value, ^d^ The effect of two‐way factor interactions on System Usability Scale Items
